# Words on fire: Nurses’ communication attitudes in the burnout equation

**DOI:** 10.1371/journal.pone.0358785

**Published:** 2026-09-24

**Authors:** María del Carmen Giménez-Espert, Lucía Sanchis-Giménez, Vicente Javier Prado-Gascó

**Affiliations:** 1 Department of Nursing, Faculty of Nursing and Chiropody, University of Valencia, Valencia, Spain; 2 Faculty of Psychology and Speech Therapy, University of Valencia, Valencia, Spain; 3 Department of Social Psychology, Faculty of Psychology and Speech Therapy, University of Valencia, Valencia, Spain; Universiti Sains Malaysia - Kampus Kesihatan, MALAYSIA

## Abstract

Nursing professionals are exposed to stressful situations. One of them could be related to how they communicate with patients and their families. However, literature on the role of communication in occupational stress is scarce. There seems to be no consensus as to whether communication is a job demand, a consequence of stress or could even act as a mediator between the stress process. The aim of this study was to discuss the role of attitudes towards communication with the patient in the stress process in nurses. The sample consisted of 193 nurses recruited from four public hospitals in the Valencian Community. They were previously informed about the aim of the study. Job demands and resources, burnout and attitudes towards communication with the patient were evaluated with the UNIPSICO Battery, Burnout Syndrome Evaluation Questionnaire (CESQT) and the reduced version of the Attitudes Towards Communication Questionnaire (ACO-R), respectively. Three hypothesized models were evaluated with PLS-SEM. The results of the analysis showed that attitudes towards communication did not influence burnout. Nor did they mediate the stress process. Attitudes towards communication were a consequence of burnout caused by the imbalance between job demands and resources. Thus, when nurses suffer from burnout due to exposure to poor working conditions, their attitudes towards communication may deteriorate.

## Introduction

Healthcare professionals are often exposed to stressful working conditions, which can negatively impact their physical and mental health [1]. According to the Employment and Social Developments in Europe (ESDE) review, health-related professions had much higher than average exposure to physical and emotional demands. Specifically, in 2021, nurses were the highest-ranked occupation reporting problems at work, experiencing job strain in 61% of the cases, double the 30% average in other job categories [2]. Additionally, the OECD (2024) showed different data from various countries [3]. For example, the Employee Survey (WNE) conducted in the Netherlands found that only 35% of the healthcare professionals felt they had sufficient time to properly pay attention to their patients. Meanwhile, in Belgium, 28.2% of health workers indicated a score of 7 or more out of 10 when asked if they would leave their jobs. Similar data, even more problematic, were found in the case of Spain. For example, regarding time pressure exposure, 45.2% of the professionals related to healthcare and social services’ sectors reported being exposed to time pressure at work, affecting their mental health. This figure was higher than in other job categories, such as administrative sector or other services (ranging from 27.1–28.4%) [4]. These types of problems could lead to enhance the intention to leave the profession. Specifically, 39.4% of the nurses said they intended to leave the job at some point within the next 10 years and 92.7% of the respondents cited working conditions as the main cause [5].

All these stressful conditions are due in part to the so-called psychosocial risks. The European Agency for Safety and Health at Work (2014) defined psychosocial risks as work aspects related to its design and the social, organizational and work management contexts that can cause both physical and psychological damage [6], conceptualized by literature as job demands and resources [7]. Beyond the prevalence itself, how can psychosocial risks affect workers and organizations?

Regarding organizations, these risks seem to be associated with high rates of personnel turnover [8] and elevated levels of presenteeism [9], more errors at work [10], a higher number of accidents at work [11], all of which can affect business productivity. As for the workers, psychosocial risks could negatively impact their health [12], leading to the appearance of burnout syndrome [13], occupational fatigue [14], low levels of work engagement [15] and even health conditions such as cognitive failures [16] or musculoskeletal disorders [17], for example.

These negative effects can occur since psychosocial risks can lead to high stressful working conditions. Different theoretical models aim to explain this phenomenon. The Karasek demand-control model (1979) is an example, which establishes that occupational stress is caused by a mismatch between the demands at work and the control workers have [7]. Other models that fit better to the aim of this study might be the Job Demand-Resources (JD-R), formulated by Bakker and Demerouti (2007) [18] or the UNIPSICO model by Gil-Monte (2014) [7], with the latter being an extension of the JD-R model. According to both theoretical models, stress appears when there is an imbalance between job demands and resources, causing burnout syndrome. In addition, the UNIPSICO model considers other consequences of a prolonged exposure to demands at work and a lack of resources, like absenteeism or low job satisfaction. This study was based on these two occupational stress models.

Among the job demands that seemed to be positively related to burnout, the ones that most stand out in literature were interpersonal conflicts [19], lack of organizational justice or inequity [20], role dysfunctions (role conflict and/or lack of role clarity) [21,22], workload [23], workplace violence [24]. Meanwhile, the most common resources that could contribute to reduce burnout were social support at work [25], autonomy [26] and resources provided by the organization [27].

Although every professional sector is exposed to occupational stress due to precarious working conditions, nurses are one of the most affected ones [28,29]. This situation is particularly concerning considering that nurses play a key role in health systems, being one of the agents who interact most with patients.

In addition to the common demands encountered by workers, nursing professionals find themselves facing stressful situations inherent to their profession, such as being responsible for the health of others [30], covering staff shortages and shift work [31].

Another stressful situation nurses need to face could be communication with patients and their families [32]. Interactions with patients seem to be the cornerstone of the nurse-patient relationship, being the most important part of nurses’ work [33]. Studies demonstrate that effective communication has multiple benefits both to patients and nursing professionals. For example, as for the patients, evidence showed a positive association between effective communication and quality care [34]. Meanwhile, as for the professionals, positive communication appears to be positively associated with a better performance and job satisfaction in work challenges [35].

However, despite the importance of communication in nursing work, the majority of studies has focused on communication in specific situations, such as cancer patients [36] or breaking bad news communication [37], ignoring communication in general. This lack of articles is also reflected in the few assessment instruments that exist, since communication with the patient measurement is complex. In this sense, as literature suggests, attitudes towards communication seem to be the best predictor of communication with the patient [33]. Therefore, the need to develop a tool that would assess attitudes towards communication with the patient in clinical practice arose, resulting in the ACO instrument. A questionnaire which evaluates communication with the patient through the attitude components (affective, cognitive and behavioral) [33].

Furthermore, few studies focused on the role of communication in occupational stress.

In this regard, some questions arise. *Is communication another demand at work? Or is it something that can be affected by job stress, that is, a consequence? Or even can communication affect the stress process, not being a job demand but a possible mediating variable in the indirect effects of job demands and resources on burnout?*

After reviewing the literature, there seems to be no consensus about it. It has been impossible to find any study that approaches the specific role communication with the patient plays in the traditional stress models. However, some articles did seem to explore the nature of this variable, which allowed us to hypothesize its possible association to the stress process.

In this sense, some studies showed that communication problems could be one of the most relevant stressors in nurses [38] affecting burnout levels or stress in general and being considered another job demand. Works such as that by Saravanan et al. (2023) found that some of the contributors to stress expressed by nurses were based in admission processes and communication tasks [39]. From this perspective, communication might be considered as another contributor to negative consequences at work like burnout, along with the traditional job demands and the insufficiency of resources.

In contrast, even though there is no literature focused on communication with the patient in the stress process, beyond communication technology [40], some studies emphasized how stress or burnout seemed to affect how nurses perform at work [18], being communication tasks part of their job. For example, there are some studies that focus on how stress can affect communication with families or colleagues at work [41]. In line to this, studies such as Khodabandeh-Shahraki et al. (2019) suggested that job stress was a barrier to effective communication [42].

Lastly, even though we have not been able to find any study that focus on the possibility that communication is a mediator in the job demands and resources-burnout relationship, some literature based on JD-R and UNIPSICO models suggests that stress process could be affected by organizational aspects, like organizational support [43], but also by intrinsic characteristics of the individual, such as personality traits [44], coping strategies [45] or even the attitudes workers have towards their job and what they do [46]. From this point of view, despite the lack of literature, attitudes towards one of the principal tasks in nursing work, communication with the patient, were expected to intervene in the impact of job demands and resources on burnout.

Therefore, as has been exposed, all professional sectors are affected by poor working conditions, but it is especially problematic in the case of nurses. One of the stressful situation nurses face at work is communication with the patient. However, most of the literature has focused on concrete contexts like breaking bad news and cancer context and has ignored nurses’ communication in general, even more the attitudes towards communication. Consequently, there is no literature about the association between communication in general and occupational stress, not being clear the role of communication in the job stress model and its principal consequences, especially burnout.

**Fig 1:**
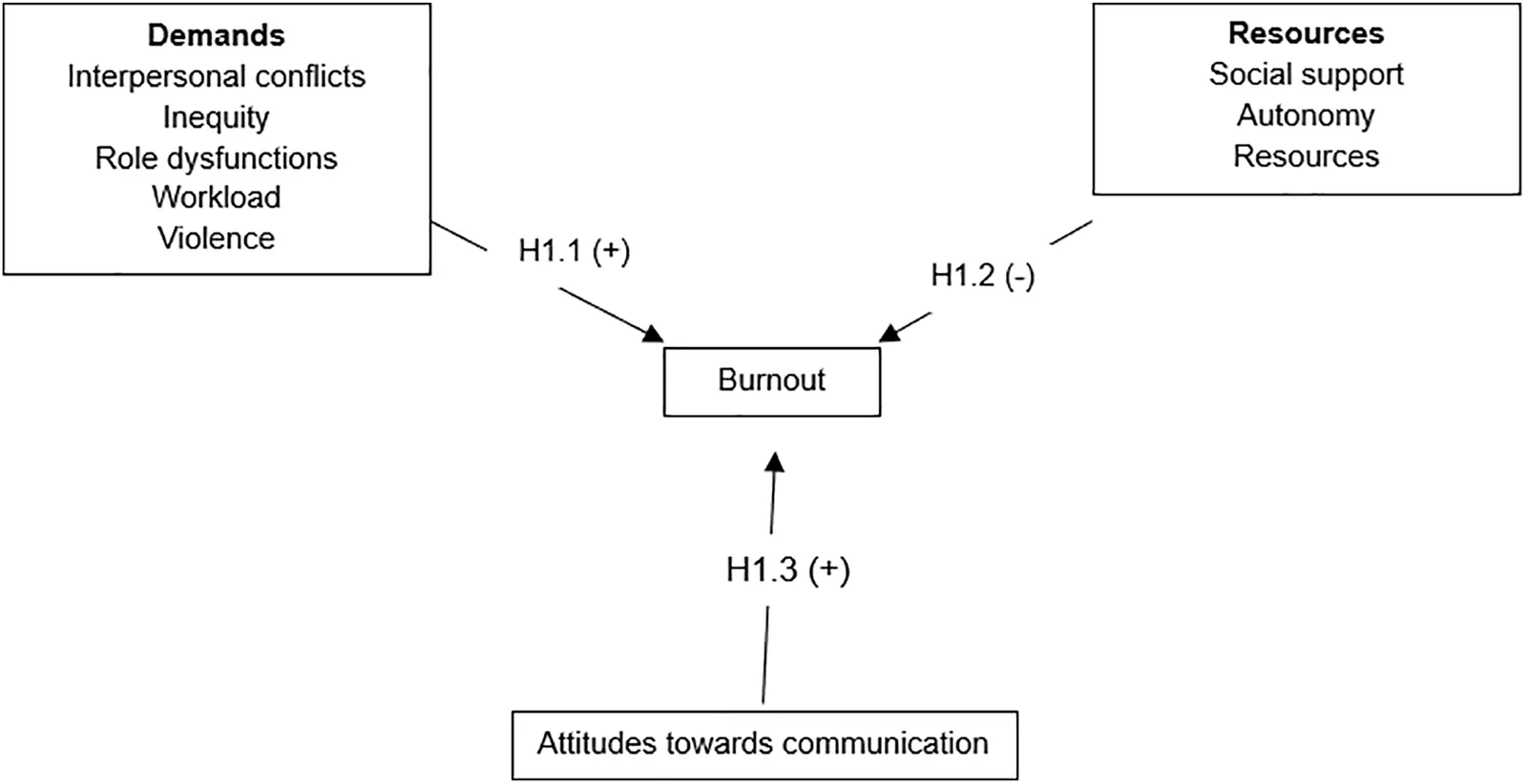
First hypothesized model.

**Fig 2:**
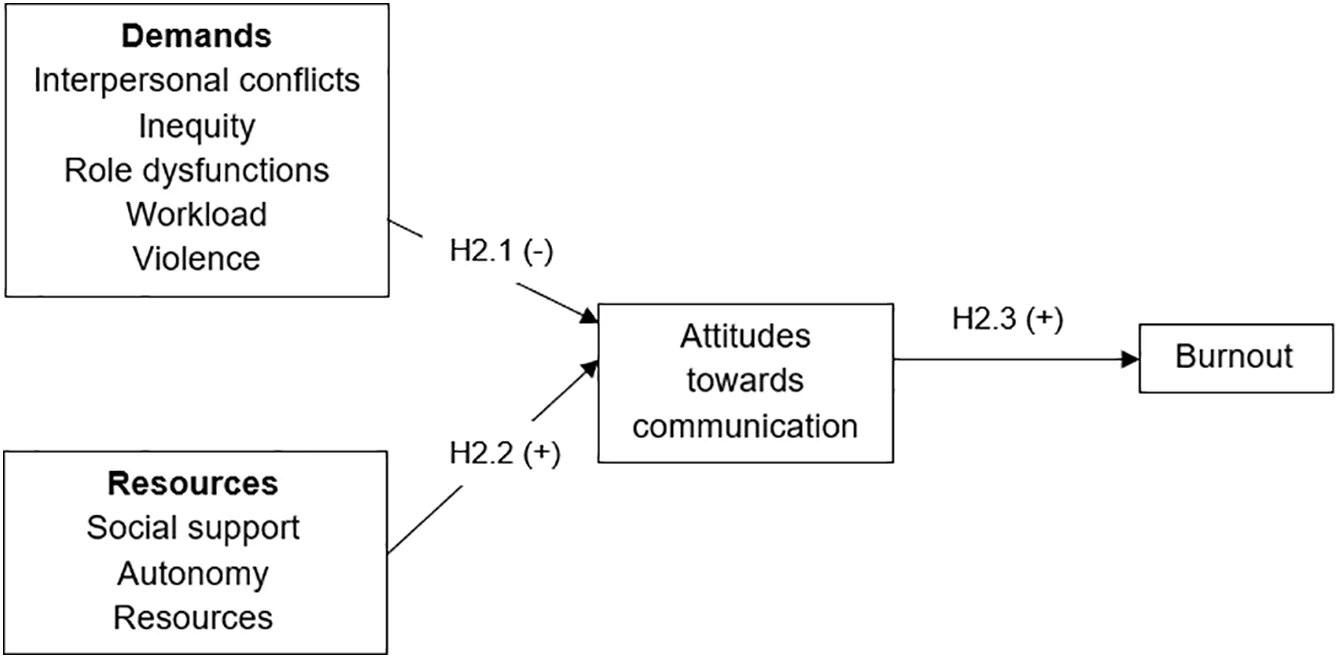
Second hypothesized model.

**Fig 3:**
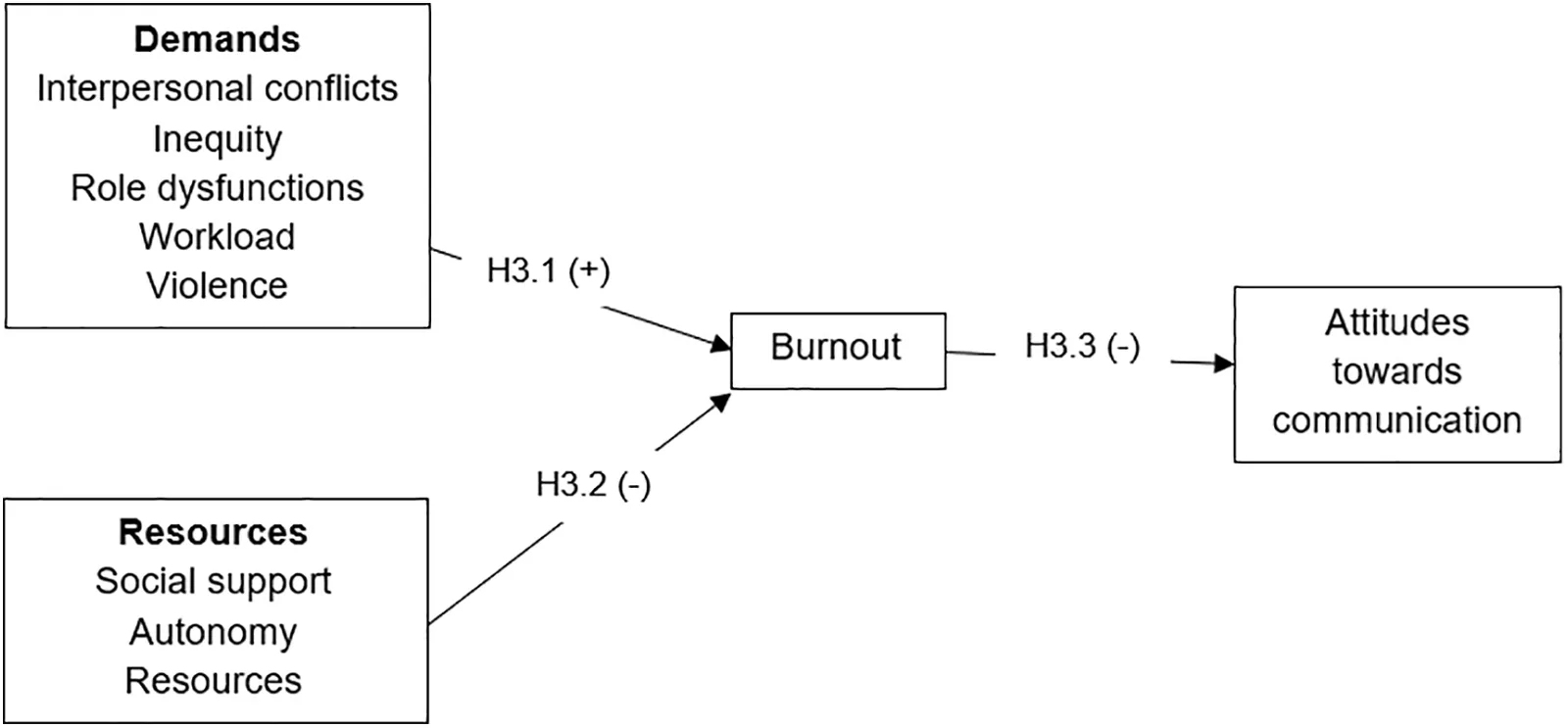
Third hypothesized model.

So, the present study addresses a critical gap in the literature. This study aims to analyze the role of the attitudes towards communication with the patient in the process of job stress in nurses, emerging the following hypothesis:

H1: Attitudes towards communication could be a job demand in stress process (Fig 1).
H2: Attitudes towards communication could affect the job demands and resources-burnout relationship as a statistical mediator (Fig 2).
H3: Attitudes towards communication could be a consequence of burnout, which at the same time is a consequence of job demands and resources (Fig 3).

## Materials and methods

### Design

This was a cross-sectional and correlational study.

### Participants

A total of 193 nurses participated in the present study, 88.5% of which were women. Their ages ranged from 21 to 64 years (*M* = 41.56; *SD* = 10.15). Regarding their care experience, the average was 16.51 years (*SD* = 10.46), ranged from 1.08 years to 43.92 years. All of the nurses belonged to 4 public hospitals in the Valencian Community, Spain. As for their education level, 85.3% of the nurses were graduates and 14.7% had a master’s degree. Then, in relation to their work situation, 17.4% of the professionals had a temporary contract, 55.3% had an interim contract, 26.8% had a permanent position and 0.5% reported having another type of contract. As for their shift pattern, 69.9% of the nurses indicated having rotating shift work while 29.6% had morning shift. Lastly, regarding the department/specialty, 23.9% of the nurses belonged to internal medicine department, 11.4% to pediatrics, 10.3% to emergencies department, 9.2% to intensive care unit (ICU), 4.3% to oncology department and another 4.3% belonged to operating room.

Considering a population of 22,000 nurses in the Valencian Community, a confidence level of 95% and an alpha error of 8%, the estimated minimum sample size was calculated, yielding a recommended minimum sample size of 150 participants. The number of participants included in the study was higher than this minimum requirement.

### Instruments

In this study, psychosocial risks were grouped into two categories: job demands and resources. Additionally, burnout and attitudes towards communication were also evaluated. All instruments presented adequate psychometric properties in previous studies [47–49], something that can also be observed in the present work.

#### Demands.

The majority of the demands was examined with the UNIPSICO battery [50]. All scales were Likert type with 5 response options, being 0 = never and 4 = very frequently: every day. The higher the score, the higher the levels of demand. The demands were:

- **Interpersonal conflicts**: it refers to the existence of conflicts with other nurses, patients and their families. This scale consists of 6 items (e.g., How often do you have conflicts with patients?). In the present study, it has also shown adequate psychometric properties [Cronbach’s alpha (α) = 0.75; Composite Reliability (CR) = 0.83; Average Variance Extracted (AVE) = 0.45].
- **Inequity**: it refers to the perception of lack of organizational justice. This scale consists of 6 items (e.g., I don’t get much reward for the efforts I put into my work), with one reversed (α = 0.79; CR = 0.85; AVE = 0.52).
- **Role conflict**: it refers to the situations in which the professional has to respond to incompatible instructions. This scale consists of 4 items (e.g., I am asked to perform functions and tasks for which I am not authorized) (α = 0.68; CR = 0.81; AVE = 0.51).
- **Role clarity**: it refers to the situations in which the professional is clear about what is expected of him or her, inverse to the demand of role ambiguity. This scale consists of 5 items (e.g., I know what my responsibilities are at work) (α = 0.82; CR = 0.87; AVE = 0.58).
- **Workload**: it refers to the perception of both quantitative and qualitative work overload. This scale consists of 6 items (e.g., Do you think you have to do a job that is too difficult for you?) (α = 0.62; CR = 0.76; AVE = 0.35).

The violence demand was measured with an adaptation of a workplace violence questionnaire [51]. It was Likert type with 5 response options, being 1 = strongly disagree and 5 = strongly agree. The higher the score, the higher the levels of workplace violence. It consists of 6 items divided in 3 types of workplace violence: (1) Physical violence: it refers to beating, kicking, pushing or pulling (e.g., In the preceding 12 months, you have experienced an increase in physical violence (e.g., beating, kicking, pushing or pulling) from patients, patients’ families or visitors.). (2) Verbal violence: it refers to abusive language, verbal harassment or cynical comments (e.g., In the preceding 12 months, you have experienced an increase in verbal violence (e.g., abusive language, verbal harassment or cynical comments) from patients, patients’ families or visitors.). (3) Psychological violence: it refers to threats, intimidation, discrimination, exclusion and harassment (e.g., In the preceding 12 months, you have experienced an increase in psychological violence (e.g., threats, intimidation, discrimination, exclusion and harassment) from patients, patients’ families or visitors.). All types of workplace violence were asked both from patients, patients’ families or visitors and from colleagues, supervisors or managers. This questionnaire also showed adequate psychometric properties in this study (α = 0.83; CR = 0.87; AVE = 0.53).

#### Resources.

The resources were evaluated with the UNIPSICO Battery [50] with three scales, all of which were Likert type with 5 response options, being 0 = never and 4 = very frequently: every day. The higher the score, the higher the levels of the resource. The resources were:

- **Social support:** it refers to the support nurses perceive they have from colleagues and superiors. This scale consists of 6 items (e.g., Do you feel appreciated at work by the center’s management?) (α = 0.75; CR = 0.83; AVE = 0.45).
- **Autonomy:** it refers to the degree of autonomy nurses perceive to manage their work. This scale consists of 5 items (e.g., In this job I have the independence to decide how to do it.) (α = 0.73; CR = 0.78; AVE = 0.43).
- **Resources:** it refers to the resources nurses perceive the organization provides. This scale consists of 8 items (e.g., I have the necessary resources to work.) with one reversed (α = 0.75; CR = 0.82; AVE = 0.37).

#### Burnout.

Burnout was measured with a reduced version of the Burnout Syndrome Evaluation Questionnaire (CESQT) [52] of 7 items, which was prepared by the research team with prior approval from the author. This scale was Likert type with 5 response options, being 0 = never and 4 = very frequently: every day. The higher the score, the higher the levels of burnout. The original instrument consisted of 20 items divided in 4 dimensions: (1) Excitement about work: it refers to the enthusiasm nurses feel for work (e.g., I feel excited about my job.), being evaluated inversely, the higher score, the lower levels of burnout. (2) Emotional exhaustion: it refers to feeling exhausted at work, leading to poor effectiveness (e.g., I feel overwhelmed by my job.). (3) Indolence: it refers to cynicism towards work (e.g., I don’t feel like seeing some patients.). (4) Guilt: it refers to the appearance of regrets because of nurses’ performance at work (e.g., I feel guilty for some of my attitudes at work) (α = 0.68; CR = 0.79; AVE = 0.36).

#### Attitudes towards communication.

Attitudes towards communication were evaluated with the reduced version of the Attitudes Towards Communication questionnaire (ACO-R) [53] of 12 items. This scale was Likert type with 5 response options, being 1 = strongly disagree and 5 = strongly agree. The higher the score, the higher the positive attitudes towards communication. The original instrument was elaborated by Giménez-Espert and Prado-Gascó (2018) (intellectual property registered at the University of Valencia on 8 April 2019, registration number: UV-MET-201917R) [33]. It consisted of 25 items divided in 3 dimensions: (1) Affective dimension: it refers to situations that can generate anxiety in nurses (e.g., I am nervous when I explain the procedure I am going to perform to the patient and/or family member.), being evaluated inversely, the higher score, the lower positive attitudes towards communication. (2) Cognitive dimension: it refers to the importance for nurses of aspects related to the orientation in the unit, information about the recovery, information on discharge care and to the collaboration with other colleagues (e.g., It is necessary to inform the patient and/or family about how they can help in the recovery.). (3) Behavioral dimension: it refers to how nurses act with respect to the patient and/or family (e.g., I usually reinforce and clarify information to the patient and/or family to obtain informed consent.) (α = 0.83; CR = 0.86; AVE = 0.34).

### Procedure

Data collection was carried out through convenience sampling. Direct patient care nurses were recruited from four public hospitals in the Valencian Community. The inclusion criteria were being actively employed at the selected hospitals and having provided their informed consent to participate in the study. Researchers reached out to the centers via e-mail and, once they received the Ethics Committees for Clinical Research from each hospital, contacted the Directors of Nursing and supervisors. The same cohort completed the assessment instrument at one time. Nurses who agreed to participate were given 2 months to complete the paper questionnaire. To improve the response rate, follow-up reminder emails were distributed two weeks after the initial mailing. Given the low response rate, expanding the data collection period was necessary in some cases. So, the data were collected during the period from December 2022 and September 2024. The time of completion of the instrument was approximately 35 min.

The authors had permission from Professor Gil-Monte to use the UNIPSICO battery and Burnout Syndrome Evaluation Questionnaire (CESQT). Permission from the authors of the ACO-R instrument was also provided as two of the authors are authors of the present work as well.

Since all data were collected at a single point in time from the same source, common method bias (CMB) risk was present. So, to overcome CMB, we have examined it by two approaches: from a procedural point of view, we aimed to reduce participants’ desirability by indicating that there were no right or wrong answers to the items in the questionnaire and eliminate common scale properties like scale type, as UNIPSICO battery went from 0–4 and ACO instrument from 1–5 [54]. Meanwhile, from the statistical point of view, we assessed CMB using the full collinearity test in SmartPLS, widely used in the PLS methodology literature [55].

### Data analysis

Descriptive analyses were conducted with SPSS Statistics 27 software (IBM Corporation, Armonk, NY, USA). On the other hand, to evaluate the three hypothesized models, Partial Least Squares Structural Equation Models (PLS-SEM) were carried out. In comparison to CB-SEM, PLS-SEM was chosen due to its prediction-oriented nature, its robustness and its suitability for exploratory complex models [56]. First, missing data was handled using pairwise deletion. Then, potential common method bias (CMB) was examined through the variance inflation factor (VIF) values of the inner or structural models [57]. And finally, measurement and structural models were evaluated, assuming them as reflective models [58]. Both analyses were performed with the software SmartPLS v.4.1.1.3 [59].

## Results

First, the descriptive analysis of the variables are presented in Table 1.

**Table 1:** Descriptive analysis.

Variables	Mean	SD^a^	Min.^b^	Max.^c^	Range
Interpersonal conflicts	0.51	0.47	0.00	3.00	0-4
Inequity	1.88	0.81	0.00	4.00	0-4
Role conflict	1.06	0.76	0.00	3.00	0-4
Role clarity	3.02	0.74	0.80	4.00	0-4
Workload	2.08	0.60	0.50	3.67	0-4
Violence	1.84	0.77	1.00	5.00	1-5
Social support	2.72	0.73	0.17	4.00	0-4
Autonomy	2.31	0.72	0.20	4.00	0-4
Resources	2.48	0.67	0.38	4.00	0-4
Burnout	1.06	0.61	0.00	2.86	0-4
Attitudes towards communication	4.43	0.56	2.33	5.00	1-5

^a^SD: standard deviation.

^b^min.: minimum.

^c^max.: maximum.

Then, the different models under study were evaluated. Before analyzing the measurement and structural models, common method bias (CMB) was assessed using the full collinearity test in SmartPLS, widely used in the PLS methodology literature [55]. According to Kock (2015), when all the variation inflation factor (VIF) values of the inner or structural models are lower than 3.3, the model can be considered free from common method bias [57]. In this case, all of the VIF values of the three structural models were lower than the cut-off point.

Once the common method bias (CMB) was tested, the measurement models were analyzed, for which internal consistency, convergent validity and discriminant validity were observed. These measurement models were assumed as reflective models [58].

The internal consistency was examined with Cronbach’s alpha and composite reliability (CR). Most of the values were found above the cut-off point of 0.70 [60]. As for the convergent validity, the average variance extracted (AVE) index was calculated, most of which were higher than the reference value of 0.50 [60]. Regarding the variables which did not reach the established cut-off point, they all were considered maintaining the original internal structure because of their CR values, as Fornell & Larcker (1981) suggested [61]. These authors indicated that measurement models whose AVE was between 0.40 and 0.49 were considered acceptable as long as their CR was greater than 0.60, as it occurred in workload or burnout cases when rounded to the nearest tenth. So, convergent validity was established and it was not necessary to modify the scales structure. Other published works also followed this methodological recommendation [62]. All of these indicators are presented in the instruments’ section.

Finally, regarding discriminant validity, the heterotrait-monotrait correlation ratio (HTMT) was carried out. All values were below 0.85 and the 90% bootstrap confidence interval did not include the value one [63], so discriminant validity was confirmed. All of these values were presented in the instruments section.

After examining the measure models, the three structural models were evaluated. The first model considered ACO as another demand while the second model considered ACO as a mediator between job demands and resources-burnout relationship. The last model considered ACO as a consequence of burnout, being burnout a consequence of an imbalance between job demands and resources.

To evaluate the three structural models, goodness of fit was examined with the standardized root mean square residual values (SRMR), establishing all SRMR values should be below the cut-off point of.08 according to Hu and Bentler (1999) [64]. Also, as SRMR indicators seemed to present some limitations in the PLS-SEM context, d_ULS and d_G values were carried out, which had to be below the 95% bootstrap-based quantiles to indicate an acceptable model fit [60]. Lastly, the significance of levels of the path coefficients and the determination coefficients *R*^*2*^ were observed, both generated with the bootstrapping technique of 5000 samples. Following Hair et al. (2022) guidelines, this significance was analysed through the boostrap confidence intervals, which should not contain the value zero for the effects to be considered significant [60].

### As a demand

**Fig 4:**
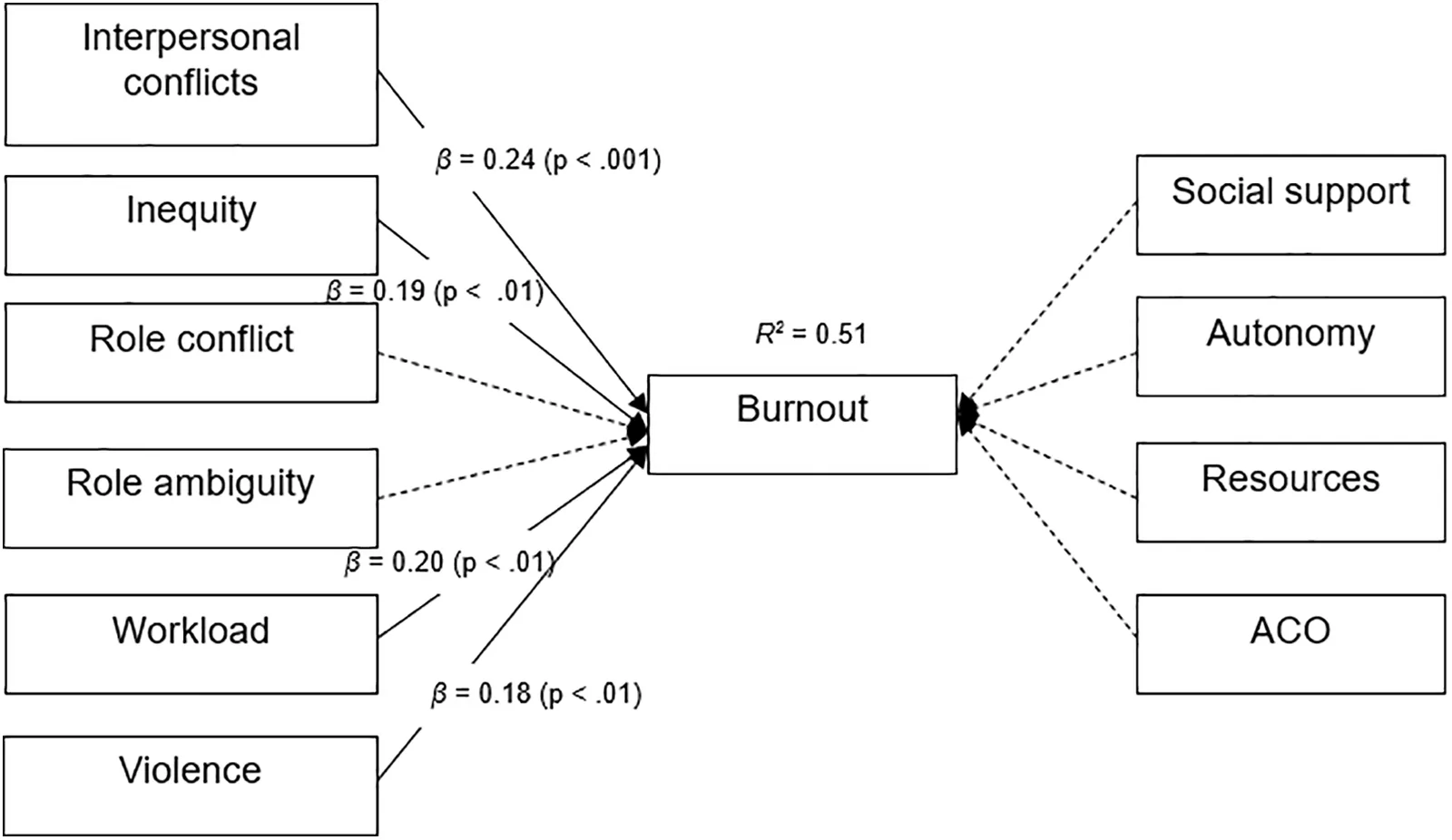
Results of the first hypothesized model.

The results obtained for the first model are shown in Fig 4. First, SRMR value was 0.09. Although this value was above 0.08, some authors consider a value below.10 could be accepted in complex models [63]. Observing d_ULS and d_G, both indicated an acceptable model fit.

Based on the results, burnout was explained by 50.6% of the variance. As for the antecedent variables, the only demands that turnout out to be influencing burnout were interpersonal conflicts (*β* = 0.24, *t* = 4.08, *p* < .001), inequity (*β* = 0.19, *t* = 3.02, *p* < .01), workload (*β* = 0.20, *t* = 3.28, *p* < .01) and violence (*β* = 0.18, *t* = 2.83, *p* < .01). As for *t*he resources, none of them were found *t*o significantly influence burnout. ACO was also not a significant antecedent of burnout.

### Indirect effects of antecedents on burnout through ACO

In this second model, SRMR value was 0.09, so goodness of fit was acceptable [63]. Observing d_ULS and d_G, both indicated an acceptable model fit.

According to Hair et al. (2022), the analysis of the mediation begins with the evaluation of the measurement models [60]. All reliability and validity indicators were included in the Instruments section. Then, structural model has to be assessed by examining path coefficients or the direct effects of the antecedent variables (job demands and resources) on burnout. Direct effects are shown in Table 2. Final step consists of bootstrapping indirect effects values of the antecedent variables on burnout (Table 3).

**Table 2:** Direct effects of antecedent variables (job demands and resources) on burnout.

Path	*β*	Mean	SD^b^	*t* value
Interpersonal conflicts > burnout	0.23	0.24	0.06	3.84^***c^
Inequity > burnout	0.19	0.19	0.07	2.89^**d^
Role conflict > burnout	0.10	0.11	0.08	1.40
Role clarity > burnout	−0.05	−0.04	0.08	0.61
Workload > burnout	0.19	0.20	0.06	3.03^**^
Violence > burnout	0.19	0.18	0.07	2.83^**^
Social support > burnout	−0.01	−0.01	0.07	0.10
Autonomy > burnout	0.13	0.12	0.08	1.68
Resources > burnout	−0.16	−0.16	0.11	1.48

^a^ACO: attitudes towards communication.

^b^SD: standard deviation.

^c^*** : *p* < .001.

^d^** : *p* < .01.

**Table 3:** Indirect effects of antecedent variables (job demands and resources) on burnout.

Path	*β*	Mean	SD^b^	*t* value
Interpersonal conflicts > ACO > burnout	0.00	−0.00	0.01	0.02
Inequity > ACO > burnout	0.00	0.00	0.01	0.07
Role conflict > ACO > burnout	−0.00	−0.00	0.01	0.04
Role clarity > ACO > burnout	−0.02	−0.02	0.02	0.83
Workload > ACO > burnout	−0.00	−0.00	0.01	0.13
Violence > ACO > burnout	0.00	0.00	0.01	0.04
Social support > ACO > burnout	−0.00	−0.00	0.01	0.30
Autonomy > ACO > burnout	−0.01	−0.01	0.02	0.51
Resources > ACO > burnout	−0.01	−0.01	0.02	0.75

^a^ACO: attitudes towards communication.

^b^SD: standard deviation.

So, as for the path coefficients or direct effect of the antecedent variables, same demands significantly influenced burnout as in the first hypothesized model, being interpersonal conflicts (*β* = 0.23, *t* = 3.84, *p* < .001), inequity (*β* = 0.19, *t* = 2.89, *p* < .01), workload (*β* = 0.19, *t* = 3.03, *p* < .01) and violence (*β* = 0.19, *t* = 2.83, *p* < .01). None of *t*he resources turned ou*t* to have a significant impact on burnout. However, none of the antecedent variables showed significant indirect effects on burnout through ACO as a mediator. Therefore, there seemed to be no statistical indirect effects consistent with theoretical mediation.

### As a consequence

**Fig 5:**
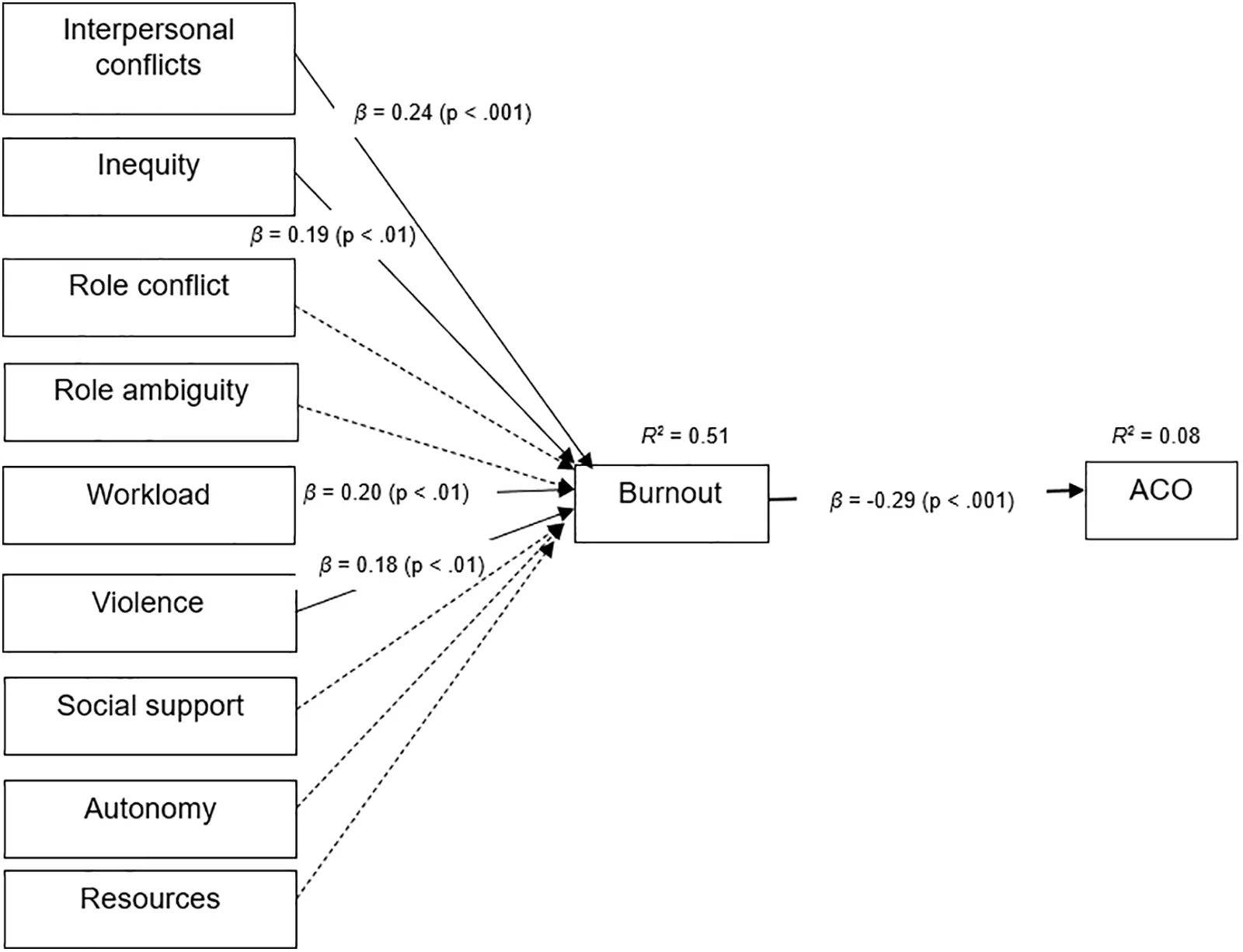
Results of the third hypothesized model.

The results obtained for the third model are shown in Fig 5. Goodness of fit was also adequate (SRMR = 0.09) [63]. Observing d_ULS and d_G, both indicated an acceptable model fit.

Burnout was explained by job demands and resources in a 49.7% of the variance. Demands that significantly influenced burnout were interpersonal conflicts (*β* = 0.24, *t* = 4.24, *p* < .001), inequity (*β* = 0.19, *t* = 2.92, *p* < .01), workload (*β* = 0.20, *t* = 3.27, *p* < .01) and violence (*β* = 0.18, *t* = 2.82, *p* < .01). As for *t*he resources, as mentioned before, none significan*t*ly influenced burnout. At the same time, burnout explained 8.3% of ACO variance (*β* = −0.29, *t* = 4.00, *p* < .001).

So, results of the three hypothesized models are collected in Table 4.

**Table 4:** Results of the three hypothesized models.

SRMR^b^	*R* ^*2*^	Path	*β*
ACO^a^ as a demand			
0.09	0.51	Interpersonal conflicts > burnout	0.24^***^
		Inequity > burnout	0.19^**^
		Workload > burnout	0.20^**^
		Violence > burnout	0.18^**^
Indirect effects of antecedents on burnout through ACO			
0.09		None of the antecedent variables exerted statistical indirect effects on burnout through ACO	
ACO as a consequence			
0.09	0.51	Interpersonal conflicts > burnout	0.24^***^
		Inequity > burnout	0.19^**^
		Workload > burnout	0.20^**^
		Violence > burnout	0.18^**^
	0.08	Burnout > ACO	−0.29^***^

^a^ACO: attitudes towards communication.

^b^SRMR: standardized root mean square residual values.

***: *p* < 0.001.

**: *p* < 0.01.

## Discussion

The scientific literature has focused on the poor working conditions nurses have and how their occupational health has been negatively impacted by these conditions [28]. This seems to be due to a mismatch between the job demands and the resources nurses, and workers in general, perceive they have [7].

The majority of the studies about nursing included the traditional demands [65–67], forgetting communication tasks as they constitute an essential part of their work [33]. After reviewing the existing literature, there seems to be no consensus on whether communication is perceived as a job demand, performing as another antecedent of burnout; a consequence of stress process considered as a result of burnout, or whether it has a mediating effect on the impact of the job demands and resources on burnout.

Therefore, regarding the importance of communication with the patient in the nursing work and its possible influence on demands and resources at work, as well as the lack of the existing literature, this study makes sense. The results obtained could guide health services’ managers in their management policies, focusing on the work aspects that seemed to influence nurses’ occupational health.

To this end, the objective of this study was to discuss the role of the attitudes towards communication with the patient (ACO) in the process of job stress in nurses. Based on this objective, three structural models emerged.

The first structural model that was hypothesized considered ACO as another job demand that could affect burnout in the same way as the traditionally studied ones. This model also included a significant negative influence of resources on burnout (H1). Data showed that ACO, or communication in general, did not seem to be perceived by nurses as a significant stressor, in contrast to some literature [38]. This could be due to the definition itself of attitude, as being considered as an internal predisposition and not an external or emotional exigence [68].

In relation to the second model, ACO did not appear to affect the impact of job demands and resources on burnout either (H2). In other words, the antecedent variables (job demands and resources) did not exert any statistical indirect effect on burnout through ACO. This could be explained by a great direct effect of the poor working conditions on burnout. In this context, the saturation of nurses’ emotional resources, originated by high demands at work, left no room for attitudes, which might lead to a non-significant effect of ACO in the stress process.

So, the results of the present study pointed to the third hypothesized model, which proposed that ACO were affected by the stress process (H3), so that a prolonged exposure to high job demands and a lack of resources could burn nurses out and once they were burned out, their attitudes and perceptions about communicating with the patient could be negatively affected or even deteriorated. Data showed that ACO was significantly affected by burnout, as literature suggested [42]. With regard to the rest of the antecedent variables of burnout, some of the job demands did not seem to exert a significant influence. This was the case for role dysfunctions (role conflict and role clarity). This could be due to the highly structured type of work, considering role clarity, and the nonexistence of role conflicts.

In the case of resources, in contrast to the majority of articles [69], none of them seemed to have a significant effect on burnout. Some studies found similar results. For example, as for the association between social support and burnout, Kiefer et al. (2025) observed no relationship between any type of social support and emotional exhaustion and cynicism, in this case indolence, despite the association with personal accomplishment, in this case excitement at work [70]. So, a non-existing relationship between social support and burnout could be explained because it has been evaluated burnout as a unique dimension. On the other hand, the non-significant impact of autonomy could be because nurses did not perceive they had enough autonomy to manage their work taking into account the numerous standardized procedures, as was mentioned before. Lastly, this sample of nurses appeared to be so burned out that they got used to such precarious conditions, resulting in a non-significant influence of the resources provided by the health services on their health [18]. All of this could generate high levels of stress, which, if prolonged over time, can lead to burnout and potentially deteriorate ACO.

So, when discussing the role of ACO in the occupational stress process, ACO seemed to be something that could be affected by burnout. So, it is when professionals suffer from burnout that their attitudes towards communication with patients and their families could change, gradually deteriorate.

This study has important contributions regarding the non-existing consensus about the role communication plays in the occupational stress process. Nevertheless, despite the contributions, it also has some limitations. For example, it is difficult to generalize the results obtained because of the non-probabilistic sampling used. Another factor potentially limiting generalizability could be the reduced scope of the sampling, as it only contemplated nurses belonging to 4 hospitals in one Spanish region. Given the post hoc nature of the sample size assessment, the results should be interpreted with caution, although the sample size was representative. To overcome the sample size limitations, path coefficients and confidence intervals were given, as well as specific indicators of PLS-SEM methodology like bootstrapping settings. As for the characteristics of the sample, an imbalance was found in gender, something inherent to the job. On the other hand, data collection was through self-administered questionnaires, although this is a common method in health and social sciences investigations. This type of evaluation could introduce some bias in the results.

In relation to measurement scales, a few psychometric properties did not fully reach conventional cut-off thresholds. However, rather than modifying or removing items strictly based on empirical criteria, the original internal structures were retained in accordance with established methodological recommendations [61]. This decision was made to prioritize and preserve the overall content validity and theoretical breadth of the instrument.

Then, even though SRMR was examined, this indicator consists of a descriptive index of model fit, so it should be interpreted with caution in PLS-SEM, as it was originally developed for covariance-based SEM and does not constitute a formal test of model fit. This limitation was overcome by performing other indicators of goodness of fit like d_ULS or d_G.

And lastly, the study design itself could also affect establishing causal relationships between variables. The cross-sectional design precludes drawing causal conclusions about the relationships among the study variables. The simultaneous measurement of antecedent variables and consequences does not allow for establishing temporal precedence, which is a key requirement for causal inference. Consequently, the observed relationships should be interpreted as statistical associations rather than evidence of causal mechanisms.

So, future research should consider probabilistic sampling for collecting data, expanding the territorial scope of the study to a national level, taking into account the gender balance, as well as the use of hetero-administered questionnaires or objective measures. Following works could also include different measurements to evaluate actual causality relationships to reduce common method bias, or even experimental designs. Moreover, future studies could consider including the different dimensions of the constructs under study, instead of a unique dimension.

Despite these limitations, the present work offered key contributions for different groups. First, it shows nurses the reality of their occupational health situation, making them see their perceptions about their work are shared experiences. Then, it fills the gap in literature about how communication with patients and their families is associated with the job stress in the healthcare context, as well as it offers verification of the psychometric properties of the instruments used. Lastly, it offers managers and politicians keys to develop intervention policies focused on interpersonal conflict management training programs, reducing workload nurses have to face with or increasing security in health services in response to the violence nurses suffer from, as they turned out to affect nurses’ occupational health, as well as training programs related to effective and efficient communication strategies even in stressful conditions.

## Supporting information

S1 Data ACOBurnout_PLS. (CSV)

S1 Appendix Supplementary material. (DOCX)
