# Supplementary material for: Words on fire: Nurses’ communication attitudes in the burnout equation
Source: PLoS One. 2026 Sep 24;21(9):e0358785. doi: 10.1371/journal.pone.0358785 (PMC13606949; doi:10.1371/journal.pone.0358785)
Supplement: Supplementary file 1 [file pone.0358785.s002.docx]

**Table 1. Heterotrait-monotrait ratio (HTMT) values for discriminant validity assessment.**

|  | **IC^a^** | **IN^b^** | **RC^c^** | **RCl^d^** | **WL^e^** | **VIO^f^** | **SS^g^** | **AU^h^** | **RS^i^** | **Burnout** | **ACO^j^** |
| --- | --- | --- | --- | --- | --- | --- | --- | --- | --- | --- | --- |
| **IC** |  |  |  |  |  |  |  |  |  |  |  |
| **IN** | 0.27^1^ |  |  |  |  |  |  |  |  |  |  |
| **RC** | 0.55 | 0.31 |  |  |  |  |  |  |  |  |  |
| **RCl** | 0.32 | 0.34 | 0.52 |  |  |  |  |  |  |  |  |
| **WL** | 0.25 | 0.32 | 0.32 | 0.25 |  |  |  |  |  |  |  |
| **VIO** | 0.31 | 0.29 | 0.39 | 0.31 | 0.33 |  |  |  |  |  |  |
| **SS** | 0.34 | 0.37 | 0.48 | 0.53 | 0.33 | 0.29 |  |  |  |  |  |
| **AU** | 0.17 | 0.24 | 0.28 | 0.40 | 0.24 | 0.18 | 0.36 |  |  |  |  |
| **RS** | 0.37 | 0.44 | 0.55 | 0.57 | 0.56 | 0.32 | 0.66 | 0.35 |  |  |  |
| **Burnout** | 0.61 | 0.53 | 0.60 | 0.45 | 0.61 | 0.55 | 0.44 | 0.20 | 0.62 |  |  |
| **ACO** | 0.23 | 0.22 | 0.27 | 0.34 | 0.27 | 0.17 | 0.24 | 0.26 | 0.35 | 0.31 |  |

^a^IC: interpersonal conflicts

^b^IN: inequity

^c^RC: role conflict

^d^RCl: role clarity

^e^WL: workload

^f^VIO: violence

^g^SS: social support

^h^AU: autonomy

^i^RS: resources

^j^ACO: attitudes towards communication

^1^Values should be below 0.85 to present adequate discriminant validity

**Table 2. Outer loadings of indicators.**

|  | **IC^a^** | **IN^b^** | **RC^c^** | **RCl^d^** | **WL^e^** | **VIO^f^** | **SS^g^** | **AU^h^** | **RS^i^** | **Burnout** | **ACO^j^** |
| --- | --- | --- | --- | --- | --- | --- | --- | --- | --- | --- | --- |
| **IC_1** | 0.53^1^ |  |  |  |  |  |  |  |  |  |  |
| **IC_2** | 0.52 |  |  |  |  |  |  |  |  |  |  |
| **IC_3** | 0.79 |  |  |  |  |  |  |  |  |  |  |
| **IC_4** | 0.70 |  |  |  |  |  |  |  |  |  |  |
| **IC_5** | 0.77 |  |  |  |  |  |  |  |  |  |  |
| **IC_6** | 0.65 |  |  |  |  |  |  |  |  |  |  |
| **IN_1** |  | 0.73 |  |  |  |  |  |  |  |  |  |
| **IN_2** |  | 0.22 |  |  |  |  |  |  |  |  |  |
| **IN_3** |  | 0.84 |  |  |  |  |  |  |  |  |  |
| **IN_4** |  | 0.70 |  |  |  |  |  |  |  |  |  |
| **IN_5** |  | 0.74 |  |  |  |  |  |  |  |  |  |
| **IN_6** |  | 0.86 |  |  |  |  |  |  |  |  |  |
| **RC_1** |  |  | 0.72 |  |  |  |  |  |  |  |  |
| **RC_2** |  |  | 0.68 |  |  |  |  |  |  |  |  |
| **RC_3** |  |  | 0.69 |  |  |  |  |  |  |  |  |
| **RC_4** |  |  | 0.78 |  |  |  |  |  |  |  |  |
| **RCl_1** |  |  |  | 0.63 |  |  |  |  |  |  |  |
| **RCl_2** |  |  |  | 0.83 |  |  |  |  |  |  |  |
| **RCl_3** |  |  |  | 0.75 |  |  |  |  |  |  |  |
| **RCl_4** |  |  |  | 0.75 |  |  |  |  |  |  |  |
| **RCl_5** |  |  |  | 0.83 |  |  |  |  |  |  |  |
| **WL_1** |  |  |  |  | 0.62 |  |  |  |  |  |  |
| **WL_2** |  |  |  |  | 0.54 |  |  |  |  |  |  |
| **WL_3** |  |  |  |  | 0.77 |  |  |  |  |  |  |
| **WL_4** |  |  |  |  | 0.43 |  |  |  |  |  |  |
| **WL_5** |  |  |  |  | 0.66 |  |  |  |  |  |  |
| **WL_6** |  |  |  |  | 0.48 |  |  |  |  |  |  |
| **VIO_1** |  |  |  |  |  | 0.75 |  |  |  |  |  |
| **VIO_2** |  |  |  |  |  | 0.83 |  |  |  |  |  |
| **VIO_3** |  |  |  |  |  | 0.89 |  |  |  |  |  |
| **VIO_4** |  |  |  |  |  | 0.60 |  |  |  |  |  |
| **VIO_5** |  |  |  |  |  | 0.62 |  |  |  |  |  |
| **VIO_6** |  |  |  |  |  | 0.64 |  |  |  |  |  |
| **SS_1** |  |  |  |  |  |  | 0.69 |  |  |  |  |
| **SS_2** |  |  |  |  |  |  | 0.74 |  |  |  |  |
| **SS_3** |  |  |  |  |  |  | 0.63 |  |  |  |  |
| **SS_4** |  |  |  |  |  |  | 0.67 |  |  |  |  |
| **SS_5** |  |  |  |  |  |  | 0.72 |  |  |  |  |
| **SS_6** |  |  |  |  |  |  | 0.56 |  |  |  |  |
| **AU_1** |  |  |  |  |  |  |  | 0.64 |  |  |  |
| **AU_2** |  |  |  |  |  |  |  | 0.71 |  |  |  |
| **AU_3** |  |  |  |  |  |  |  | 0.51 |  |  |  |
| **AU_4** |  |  |  |  |  |  |  | 0.90 |  |  |  |
| **AU_5** |  |  |  |  |  |  |  | 0.41 |  |  |  |
| **RS_1** |  |  |  |  |  |  |  |  | 0.78 |  |  |
| **RS_2** |  |  |  |  |  |  |  |  | 0.51 |  |  |
| **RS_3** |  |  |  |  |  |  |  |  | 0.63 |  |  |
| **RS_4** |  |  |  |  |  |  |  |  | 0.57 |  |  |
| **RS_5** |  |  |  |  |  |  |  |  | 0.76 |  |  |
| **RS_6** |  |  |  |  |  |  |  |  | 0.47 |  |  |
| **RS_7** |  |  |  |  |  |  |  |  | 0.39 |  |  |
| **Burnout_1** |  |  |  |  |  |  |  |  |  | 0.61 |  |
| **Burnout_2** |  |  |  |  |  |  |  |  |  | 0.57 |  |
| **Burnout_3** |  |  |  |  |  |  |  |  |  | 0.81 |  |
| **Burnout_4** |  |  |  |  |  |  |  |  |  | 0.76 |  |
| **Burnout_5** |  |  |  |  |  |  |  |  |  | 0.46 |  |
| **Burnout_6** |  |  |  |  |  |  |  |  |  | 0.41 |  |
| **Burnout_7** |  |  |  |  |  |  |  |  |  | 0.47 |  |
| **ACO_1** |  |  |  |  |  |  |  |  |  |  | 0.50 |
| **ACO_2** |  |  |  |  |  |  |  |  |  |  | 0.64 |
| **ACO_3** |  |  |  |  |  |  |  |  |  |  | 0.57 |
| **ACO_4** |  |  |  |  |  |  |  |  |  |  | 0.61 |
| **ACO_5** |  |  |  |  |  |  |  |  |  |  | 0.50 |
| **ACO_6** |  |  |  |  |  |  |  |  |  |  | 0.45 |
| **ACO_7** |  |  |  |  |  |  |  |  |  |  | 0.59 |
| **ACO_8** |  |  |  |  |  |  |  |  |  |  | 0.64 |
| **ACO_9** |  |  |  |  |  |  |  |  |  |  | 0.53 |
| **ACO_10** |  |  |  |  |  |  |  |  |  |  | 0.51 |
| **ACO_11** |  |  |  |  |  |  |  |  |  |  | 0.78 |
| **ACO_12** |  |  |  |  |  |  |  |  |  |  | 0.58 |

^a^IC: interpersonal conflicts

^b^IN: inequity

^c^RC: role conflict

^d^RCl: role clarity

^e^WL: workload

^f^VIO: violence

^g^SS: social support

^h^AU: autonomy

^i^RS: resources

^j^ACO: attitudes towards communication

^1^Outer loading values should be above 0.70 to be retained

**Table 3. Bootstrap settings (confidence intervals bias corrected) of ACO as a demand model.**

| **Path** | **Original sample** | **Sample mean** | **Bias** | **2.5%**^1^ | **97.5%** |
| --- | --- | --- | --- | --- | --- |
| Interpersonal conflicts > burnout | 0.24 | 0.24 | -0.00 | 0.13 | 0.37 |
| Inequity > burnout | 0.20 | 0.18 | -0.01 | 0.09 | 0.33 |
| Role conflict > burnout | 0.11 | 0.10 | -0.01 | -0.01 | 0.29 |
| Role clarity > burnout | -0.02 | -0.02 | 0.00 | -0.18 | 0.12 |
| Workload > burnout | 0.18 | 0.19 | 0.01 | 0.06 | 0.30 |
| Violence > burnout | 0.18 | 0.18 | -0.00 | 0.06 | 0.31 |
| Social support > burnout | 0.00 | -0.01 | -0.01 | -0.13 | 0.15 |
| Autonomy > burnout | 0.08 | 0.04 | -0.04 | -0.10 | 0.29 |
| Resources > burnout | -0.17 | -0.16 | 0.01 | -0.38 | 0.02 |
| ACO^a^ > burnout | -0.10 | -0.14 | -0.04 | -0.18 | 0.10 |

^a^ACO: attitudes towards communication

^1^Confidence intervals should not include zero for the effects to be considered significant

**Table 4. Bootstrap settings (confidence intervals bias corrected) of indirect effects of antecedents on burnout throguh ACO.**

| **Path** | **Original sample** | **Sample mean** | **Bias** | **2.5%**^1^ | **97.5%** |
| --- | --- | --- | --- | --- | --- |
| Interpersonal conflicts > ACO^a^ > burnout | 0.00 | -0.00 | -0.00 | -0.02 | 0.02 |
| Inequity > ACO > burnout | 0.00 | 0.00 | 0.00 | -0.02 | 0.03 |
| Role conflict > ACO > burnout | -0.00 | -0.00 | 0.00 | -0.03 | 0.02 |
| Role clarity > ACO > burnout | -0.02 | -0.02 | -0.00 | -0.09 | 0.01 |
| Workload > ACO > burnout | -0.00 | 0.00 | 0.00 | -0.03 | 0.02 |
| Violence > ACO > burnout | 0.00 | 0.00 | 0.00 | -0.03 | 0.02 |
| Social support > ACO > burnout | -0.00 | 0.00 | 0.00 | -0.04 | 0.02 |
| Autonomy > ACO > burnout | -0.01 | -0.01 | -0.00 | -0.05 | 0.02 |
| Resources > ACO > burnout | -0.01 | -0.01 | 0.00 | -0.07 | 0.01 |

^a^ACO: attitudes towards communication

^1^Confidence intervals should not include zero for the effects to be considered significant

**Table 5. Bootstrap settings (confidence intervals bias corrected) of ACO as consequence.**

| **Path** | **Original sample** | **Sample mean** | **Bias** | **2.5%**^1^ | **97.5%** |
| --- | --- | --- | --- | --- | --- |
| Interpersonal conflicts > burnout | 0.24 | 0.24 | -0.00 | 0.14 | 0.36 |
| Inequity > burnout | 0.19 | 0.19 | -0.01 | 0.08 | 0.33 |
| Role conflict > burnout | 0.11 | 0.10 | -0.01 | -0.02 | 0.28 |
| Role clarity > burnout | -0.05 | -0.05 | 0.00 | -0.19 | 0.10 |
| Workload > burnout | 0.18 | 0.19 | 0.01 | 0.05 | 0.29 |
| Violence > burnout | 0.18 | 0.18 | -0.00 | 0.06 | 0.31 |
| Social support > burnout | -0.00 | -0.01 | -0.01 | -0.13 | 0.14 |
| Autonomy > burnout | 0.09 | 0.05 | -0.05 | -0.07 | 0.29 |
| Resources > burnout | -0.19 | -0.18 | 0.00 | -0.39 | 0.01 |
| Burnout > ACO^a^ | -0.29 | -0.34 | -0.05 | -0.38 | 0.33 |

^a^ACO: attitudes towards communication

^1^Confidence intervals should not include zero for the effects to be considered significant

**Table 6. PLS-SEM- specific indicators of goodness of fit of the models.**

| **Indicator** | **Model** | **Original sample** | **Sample mean** | **95%** | **99%** |
| --- | --- | --- | --- | --- | --- |
| ACO^a^ as a demand | | | | | |
| d_ULS^b^ | Saturated | 21.87^1^ | 18.88 | 23.57 | 26.81 |
|  | Estimated | 21.87 | 18.88 | 23.57 | 26.79 |
| d_G^c^ | Saturated | 6.25 | n/a | 19.23 | 24.23 |
|  | Estimated | 6.25 | n/a | 19.23 | 24.23 |
| Indirect effects of antecedents on burnout through ACO | | | | | |
| d_ULS | Saturated | 20.61 | 17.53 | 21.50 | 24.36 |
|  | Estimated | 20.68 | 17.49 | 21.38 | 24.20 |
| d_G | Saturated | 6.00 | n/a | 18.92 | 23.84 |
|  | Estimated | 6.00 | n/a | 18.92 | 23.82 |
| ACO as a consequence | | | | | |
| d_ULS | Saturated | 21.78 | 18.88 | 23.57 | 26.74 |
|  | Estimated | 22.10 | 19.89 | 24.61 | 27.66 |
| d_G | Saturated | 6.23 | n/a | 19.27 | 24.22 |
|  | Estimated | 6.24 | n/a | 19.32 | 24.26 |

^a^ACO: attitudes towards communication

^b^d_ULS: squared Euclidean distance

^c^d_G: geodesic distance

^1^Indicator values should be below the 95% bootstrap-based quantiles to indicate an acceptable model fit
